# Recognizing supraventricular tachyarrhythmias in female patients: beyond palpitations to symptoms like anxiety, panic, and nausea

**DOI:** 10.3389/fpsyt.2026.1858764

**Published:** 2026-07-22

**Authors:** Noëlle Moser-van der Geest, Dagmar I. Keller, Ksenija Slankamenac

**Affiliations:** 1 Emergency Department, University Hospital Zurich, Zurich, Switzerland; 2 Klinik Gut St. Moritz, Moritz, Switzerland

**Keywords:** anxiety, nausea, palpitation, panic attack, supraventricular tachyarrhythmia

## Abstract

**Study objective:**

To investigate sex-specific differences in the clinical presentation of Supraventricular Tachycardia (SVT) in patients presenting to a tertiary care Emergency Department (ED) and to examine whether sex-related differences in management and follow-up are observed in our cohort.

**Methods:**

We conducted a retrospective cohort study of adults presenting with palpitations and diagnosed with SVT at a tertiary care ED in 2019. Diagnoses were confirmed by electrocardiogram review. The prima-ry outcome was sex-specific differences in symptom presentation. Secondary outcomes included differences in acute treatment, treatment success, follow-up recommendations and etiologic factors. Multivariable regression analyses adjusted for age, Charlson Comorbidity Index, and prior SVT treat-ment were performed.

**Results:**

Of 375 screened patients, 278 were included (45.3% women; mean age 49.5 years (SD 19) of all pa-tients). Women had a significantly higher prevalence of hypokalaemia (11.1% vs 3.3%; adjusted odds ratio [OR] 3.7 (95% CI 1.3–10.9, p=0.018)) and reported more overall symptoms (adjusted difference 0.3, 95% CI 0.03 – 0.56, p=0.027). Leading symptoms such as palpitations (OR 3.5 (95% CI 1.3–9.0, p=0.011)), nausea (OR 2.2 (95% CI 1.1–4.5, p=0.026)), and anxiety (OR 2.9 (95% CI 1.1–8.1, p=0.031)) were more frequent in women when presenting in the ED. Atrial fibrillation was the most common di-agnosis in both sexes without any significant sex-related difference in the frequency. Atrioventricular nodal reentry tachycardia was more frequent in women (12.7% vs. 5.9%, OR 2.4 (95% CI 1.01–5.8, p=0.047)), whereas atrial flutter was significantly less common in women (3.2% vs. 11.2%, OR 0.2 (95% CI 0.07–0.7, p=0.011)). No significant sex differences were observed in acute treatment, treat-ment success or follow-up.

**Conclusion:**

Women with SVT present with a higher symptom burden and a distinct symptom profile. Clinicians should maintain a high index of suspicion and ensure electrocardiographic evaluation in all patients with compatible symptoms regardless of sex, but particularly in women presenting with palpitations, nausea, or anxiety.

## 1 Introduction

In emergency department (ED) settings, supraventricular tachycardia (SVT) represents a frequently encountered condition, evidenced by approximately 50,000 annual visits in the United States (1). This cardiac arrhythmia exhibits a prevalence rate of 2.25 per 1,000 individuals and an incidence rate of 35 cases per 100,000 patient-years (2, 3). Patients with isolated SVT frequently present to an ED for the initial event and are at risk for ED re-visits due to recurrent symptoms (2). The risk of SVT is notably higher in women, with studies indicating that they represent 60.8% of palpitation-related presentations in U.S. EDs (2, 4, 5). Some sex-specific differences in SVT have already been documented in previous research. Existing studies show that AV Nodal Reentry Tachycardia (AVNRT) occurs more frequently in women, while AV Reentry Tachycardia (AVRT) or Atrial Fibrillation (AF) atrial flutter are more common in men (2, 6–8). The influence of hormonal or cyclical factors on SVT in women has been explored, with indications that most paroxysmal SVTs tend to occur during the premenstrual days (9). Additionally, literature points to a delayed diagnosis of SVT in women compared to men, often due to initial misinterpretation of symptoms as panic or anxiety disorders (4, 7, 8). Furthermore, it has been described that women are more often primarily treated with antiarrhythmic medications rather than ablation therapy (2, 7). An observed trend in clinical practice involves a protracted interval prior to the initiation of first-line ablation therapy (4–8, 10). Divergent findings are reported regarding the duration between the establishment of a diagnosis and the subsequent ablation intervention. Some studies describe an extended timeline (6), whereas others report no significant difference in the time elapsed from accurate diagnosis to therapeutic intervention (4, 10). Similarly, discrepancies are noted in the recurrence rates post-ablation. Certain research indicates an elevated recurrence rate in younger female patients (2), while other studies discern no disparity in the success rates or recurrence frequencies post-ablation (6). In general, radiofrequency ablation has been demonstrated to be equally safe and effective across sexes (7), with no discernible differences in access to healthcare resources or in health-related quality of life outcomes between male and female patients (2). To the extent of our knowledge, the exploration of sex-specific disparities in the clinical manifestation of SVT is relatively limited. Existing data indicates that women generally exhibit a higher number of symptoms compared to men (11). Variations have been observed in several parameters, including referral patterns, duration of arrhythmic episodes, socioeconomic status, and the array of symptoms presented (4). Nonetheless, these differences have not been extensively detailed specified or quantified in the literature.

Therefore, the objective of this study was to investigate potential differences in the clinical presentation of female and male patients with SVT at the ED of a tertiary care hospital. Additionally, it aimed to determine whether the previously described sex-specific variations, particularly in the context of follow-up treatment, can also be confirmed at the current hospital.

## 2 Methods

A retrospective analysis was conducted including patients who suffered primarily from palpitations and visited the ED of a tertiary care hospital between 1st January 2019 and 31st December 2019. Patients were identified through a retrospective chart review of electronic emergency department records. All patients presenting with palpitations at the ED and diagnosed with one of the following conditions were included in the study: sinus tachycardia, atrial tachycardia, focal and multifocal tachycardia, atrial fibrillation, atrial flutter, AVNRT and AVRT (Wolff-Parkinson-White Syndrome) and junctional ectopic tachycardia. All patients underwent continuous cardiac telemetry throughout their ED stay, ensuring that any transient arrhythmia, including paroxysmal SVT, was detected and documented in the medical record.

**Figure 1:**
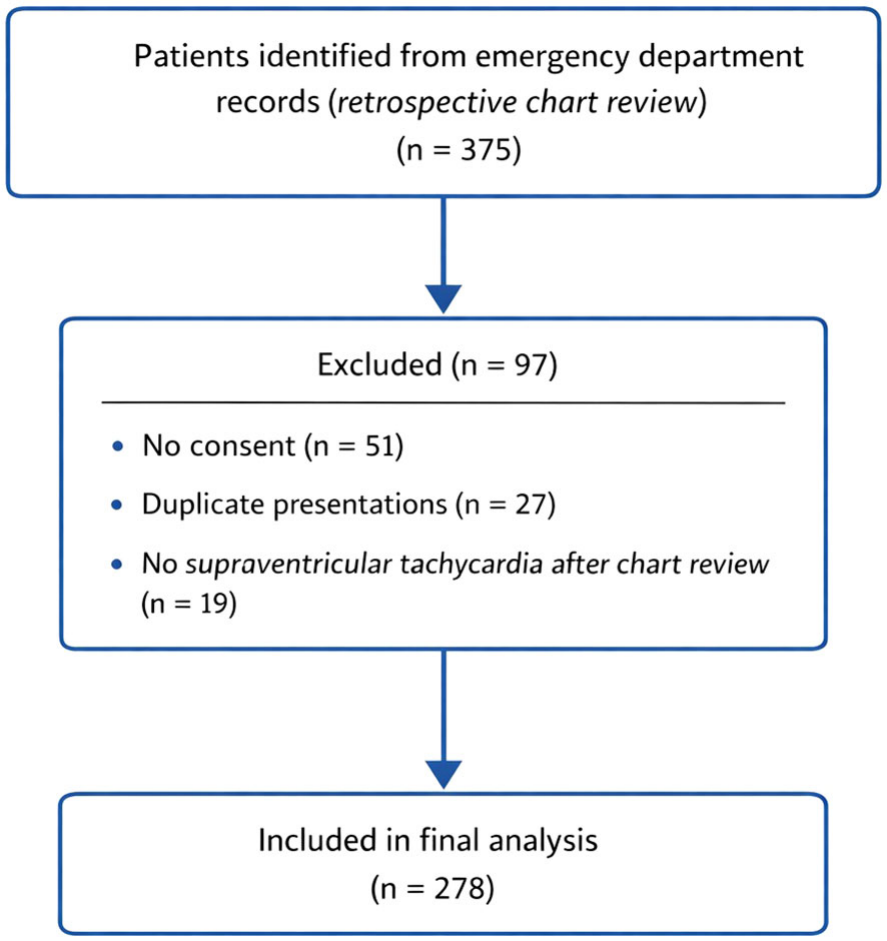
Flowchart of patient selection.

Exclusion criteria were patients presenting with normal sinus rhythm or ventricular tachycardia. Patient were also excluded due to declined general informed consent. Additionally, patients were excluded who represented twice or more often within the same year and had already been included in the study (**Figure 1**). The final diagnosis was verified by reviewing the ECGs by a cardiologist as part of the data analysis.

The study was approved by the local ethics committee.

### 2.1 Endpoints

The primary endpoint of the study was to identify any potential difference in the clinical presentation of SVT in women compared to men. For this purpose, all symptoms reported by the patients were recorded and analyzed. Secondary endpoints of the study included differences in acute therapy and its success rate, recommended follow-up treatment, and etiological factors. To this end, conversion therapies performed in the emergency setting and their effectiveness were analyzed. The recommended follow-up care documented in the medical records was also reviewed for sex-specific differences. Furthermore, risk factors for SVT were investigated to identify any sex-differences.

### 2.2 Further parameters

Examination of the respective patient records was conducted to extract specific data. This included demographic characteristics, health-related behavior, and associated medications, family history and previous treatments of SVT, the admission status of the patients, laboratory test results such as potassium, creatinine, C-reactive protein, and thyroid hormones, where hypokalemia is defined as a potassium level of <3.5 mmol/l according to laboratory guidelines. Furthermore, we assessed the diagnosis and implemented treatments including their efficacy. Additionally, information on the final diagnosis and the planned follow-up were collected. Furthermore, pre-existing comorbidities were assessed according to the validated and well-known Charlson Co-morbidity index (CCI) (12). The CCI is a tool used to categorize comorbidities based on the International Classification of Disease. Each comorbidity category is assigned a weight ranging from 1 to 6, based on the mortality risk or resource requirement associated with it. Cumulatively, each patient is assigned a score, with the probability of higher mortality and/or resource requirement positively correlating with an increasing score (12).

Participants in the study encompassed a spectrum of cases across all Emergency Severity Index (ESI) levels. The ESI is a categorization tool utilized in EDs to triage patients based on the urgency of their medical condition and the anticipated resource utilization for diagnostic or therapeutic interventions (13). The acuity is primarily gauged by the urgency of the medical condition including stability of the patient’s vital functions and the potential threat of life, limbs, or organs. Resource requirements are estimated based on the extent of medical resources anticipated to be necessary for reaching a diagnostic or therapeutic decision (13). Triage is conducted by a healthcare professional with substantial experience. ESI includes 5 levels of urgency, whereas level 1 necessitates immediate medical intervention due to a life-threatening patient condition and level 2 includes high risk situations demanding medical treatment within ten minutes (13).

### 2.3 Statistical analysis

We analyzed the distribution of variables using means and standard deviation (SD) for normally distributed data, and medians and interquartile ranges (IQR) for skewed data. We tested dataset for normality with the Kolmogorow-Smirnow test. Categorical data were presented as frequency.

The primary (number of leading symptoms) and all other secondary endpoints were compared between female and male patients using univariate and multivariable linear as well as logistic regression analysis and adjusting for some potential confounders such as age, CCI and the need for any treatment for small narrow tachycardia in the past. For all results, we reported point estimates, 95% confidence intervals and p-values (<0.05 considered significant). We performed statistical analyses using the statistical program STATA SE (version 16, Stata Corp., College Station, Texas).

## 3 Results

### 3.1 Patient characteristics

The study included a total of 278 out of an initial 375 patients. Ninety-seven people were excluded due to refusal of informed consent or repeated presentations of the same patient for identical complaints, with only the first encounter considered for inclusion. Patients were aged between 16 and 101 years, 126 (45.3%) were female and 152 (54.7%) male (**Table 1**). Across both sexes, the mean age was 49.5 years (SD 19) and 83.8% (n = 233) had a CCI < 4 (**Table 1**). The majority (84.2%) of patients presented themselves independently at the ED and 225 patients (80.9%) were triaged with an ESI 3 while 18.0% (n = 50) with an ESI 2 (**Table 2**). The median number of patients at the ED presentation was cardiopulmonary stable with normotensive blood pressure levels, good oxygen saturation and afebrile, without evidence of sex-specific differences (**Table 2**).

**Table 1:** Patient’s characteristics.

Characteristics	All patients n = 278	Male patients n = 152 (54.7%)	Female patients n = 126 (45.3%)
Age, yrs*	49.5 (19)	50 (18)	49 (20)
Charlson co-morbidity index	1 (0 – 2)	1 (0 – 2.5)	1 (0 – 2)
- < 4	233 (83.8%)	127 (83.6%)	106 (84.1%)
- ≥ 4	45 (16.2%)	25 (16.5%)	20 (15.9%)
Coronary heart disease (%)	21 (7.6%)	14 (9.2%)	7 (5.6%)
Arterial hypertension (%)	98 (35.3%)	63 (41.5%)	35 (27.8%)
Supraventricular tachycardia (%)	100 (36.0%)	57 (37.5%)	43 (34.1%)
- Age of the first event	N = 190 46.5 (34 – 61)	N = 109 50 (34 – 61)	N = 81 43 (35 – 61)
Catheter ablation in the past (%)	27 (9.7%)	15 (9.9%)	12 (9.5%)
Successful treatment in the past of supraventricular			
tachycardia (%)			
- Non required	217 (78.1%)	116 (76.3%)	101 (80.2%)
- ECV	21 (7.6%)	13 (8.6%)	8 (6.4%)
- Adenosine	10 (3.6%)	4 (2.6%)	6 (4.8%)
- EPS	10 (3.6%)	7 (4.6%)	3 (2.4%)
- Beta blockers	9 (3.2%)	9 (5.9%)	0%
- Unclear	6 (2.2%)	1 (0.7%)	5 (4.0%)
- Cordarone	3 (1.1%)	2 (1.3%)	1 (0.8%)
- Vasalva maneuver	1 (0.4%)	0%	1 (0.8%)
- Flecainide	1 (0.4%)	0%	1 (0.8%)
- modified Vasalva maneuver	0%	0%	0%
- ICD	0%	0%	0%
Cerebrovascular disease (%)	4 (1.4%)	3 (2.0%)	1 (0.8%)
Peripheral artery disease (%)	5 (1.8%)	3 (2.0%)	2 (1.6%)
Thyroid disease (%)	35 (12.6%)	16 (10.5%)	19 (15.1%)
- Medical treatment (%)	26 (9.4%)	9 (5.9%)	17 (13.5%)
Chronic obstructive pulmonary disease (%)	2 (0.7%)	2 (1.3%)	0%
Asthma (%)	15 (5.4%)	10 (6.6%)	5 (4.0%)
Diabetes mellitus (%)	27 (9.7%)	17 (11.2%)	10 (7.9%)
Chronic kidney failure (%)	22 (7.9%)	12 (7.9%)	10 (7.9%)
History of organ transplantation (%)	3 (1.1%)	2 (1.3%)	1 (0.8%)
Rheumatologic disease (%)	26 (9.4%)	12 (7.9%)	14 (11.1%)
Malignant disease (%)	7 (2.5%)	7 (4.6%)	0%
- metastatic disease (%)	2 (0.7%)	2 (1.3%)	0%
- ongoing chemotherapy (%)	3 (1.1%)	3 (2.0%)	0%
- ongoing radiotherapy (%)	0%	0%	0%
Being pregnant (%)	3 (1.1%)	0%	3 (2.4%)
Being immunosuppressed (%)	15 (5.4%)	6 (4.0%)	9 (7.1%)
Nicotine abuse (%)	52 (18.7%)	32 (21.1%)	20 (15.9%)
- in the past	43 (15.5%)	28 (18.4%)	15 (11.9%)
Regular drug abuse (%)	15 (5.4%)	9 (5.9%)	6 (4.8%)
- cocaine (%)	8 (2.9%)	6 (4.0%)	2 (1.6%)
- acute intoxication by cocaine (%)	5 (1.8%)	3 (2.0%)	2 (1.6%)
Regular alcohol intake (%)	15 (5.4%)	14 (9.2%)	1 (0.8%)
- acute alcohol intoxication (%)	6 (2.2%)	6 (4.0%)	0%
Family history positive for (%)			
- coronary heart disease	29 (10.4%)	17 (11.2%)	12 (9.5%)
- supraventricular tachycardia	3 (1.1%)	3 (2.0%)	0%
- general arrhythmia	5 (1.8%)	4 (2.6%)	1 (0.8%)

Results were presented as median (25th – 75th percentile).

ECV, Electrical cardioversion; EPS, Electrophysiological study; ICD, Implantable Cardioverter Defibrillator.

**Table 2:** Presentation during ED admission.

Parameters during ED admission	All patients n = 278	Male patients n = 152 (54.7%)	Female patients n = 126 (45.3%)
Number of self-admittances (%)	234 (84.2%)	129 (84.9%)	105 (83.3%)
Number of admittances by paramedics (%)	44 (15.8%)	23 (15.1%)	21 (16.7%)
Emergency Severity Index (ESI) (%)			
- ESI 1	0%	0%	0%
- ESI 2	50 (18.0%)	27 (17.8%)	23 (18.3%)
- ESI 3	225 (80.9%)	123 (80.9%)	102 (81.0%)
- ESI 4	3 (1.1%)	2 (1.3%)	1 (0.8%)
- ESI 5	0%	0%	0%
Presentation during (%)			
- day-shift	127 (45.7%)	79 (52.0%)	48 (38.1%)
- mid-shift	107 (38.5%)	50 (32.9%)	57 (45.2%)
- night-shift	44 (15.8%)	23 (15.1%)	21 (16.7%)
Systolic blood pressure (mmHg)	140 (128 – 155)	143 (131 – 155.5)	138 (123 – 153)
Diastolic blood pressure (mmHg)	87 (78 – 96)	89.5 (79 – 97)	84 (76 – 93)
Heart rate (/min)	92 (76 – 120)	92 (76 – 120)	93 (77 – 120)
Body temperature (°C)	36.8 (36.5 – 37.1)	36.8 (36.5 – 37.1)	36.8 (36.5 – 37.2)
Oxygen saturation (%)	98 (97 – 100)	98 (97 – 100)	98 (97 – 100)
Instable (%)	1 (0.4%)	0%	1 (0.8%)

Results were presented as median (25th – 75th percentile).

It was found that male patients were 12.7 times (95% CI 1.6 – 97.8, p=0.015) more likely to show regular overconsumption of alcohol than women (9.2% vs. 0.8%) when presenting at the ED. Conversely, women had a significantly higher prevalence of pre-medication with L-Thyroxine (11.1% vs. 4.6%; adj. OR 3.0 (95% CI 1.1 – 8.0, p=0.031), which is consistent with the fact that women more commonly suffer from hypothyroidism (14). All other patients’ characteristics are described in **Table 1**.

### 3.2 Symptoms

The majority (92.5%, n = 257) of all patients reported a sudden onset of symptoms and in nearly half of the patients (48.6%), the symptoms were self-limiting (**Table 3**). Female patients had significantly more symptoms at ED presentation than males (mean 2.3 (SD 1.1) vs. 2.0 (SD 1.1), adj. difference 0.3 (95% CI 0.03 – 0.56, p=0.027). Although all patients frequently reported palpitations, women expressed this symptom significantly more often (adj. OR 3.5 (95% CI 1.3 – 9.0, p=0.011)) at the time of ED presentation (**Table 3**). In addition, the female population reported significantly more nausea (adj. OR 2.2 (95% CI 1.1 – 4.5, p=0.026)) and anxiety (adj. 2.9 (95% CI 1.1 – 8.1, p=0.031)) during SVT compared to male patients (**Table 3**). Furthermore, a trend emerges indicating that women tend to report dyspnea as leading symptom more frequently, although the difference is not statistically significant (adj. OR 1.8 (95% CI 0.9 – 3.3, p=0.07)). In the male cohort, no symptom was notably more prevalent (**Table 3**). Other symptoms were similar across all patients including chest pain (33.1%) and dizziness (23.7%) without sex-specific differences in frequency of occurrence (**Table 3**). Less frequently mentioned symptoms are detailed in **Table 3**.

**Table 3:** Sex differences in symptoms and ED indicators.

Symptoms	Male patients n = 152 (54.7%)	Female patients n = 126 (45.3%)	Unadjusted OR (95% CI, p-value)	Adjusted OR (95% CI, p-value)
Sudden onset of symptoms (%)	137 (90.1%)	120 (95.2%)	2.2 (0.8 -5.8, p=0.12)	2.4 (0.9 – 6.7, p=0.10)
Symptoms self-limited (%)	72 (47.4%)	63 (50.0%)	1.1 (0.7 – 1.8, p=0.66)	1.0 (0.6 – 1.7, p=0.90)
First event (%)	60 (39.5%)	33 (26.2%)	0.5 (0.3 – 0.9, p=0.020)	0.5 (0.3 – 0.9, p=0.011) Resp. Male 2.0 (1.2 – 3.5, p=0.011)
Palpitation (%)	131 (86.2%)	119 (94.4%)	2.7 (1.1 – 6.6, p=0.027)	3.5 (1.3 – 9.0, p=0.011)
Nausea (%)	15 (9.9%)	24 (19.1%)	2.1 (1.1 – 4.3, p=0.031)	2.2 (1.1 – 4.5, p=0.026)
Anxiety (%)	6 (4.0%)	14 (11.1%)	3.0 (1.1 – 8.2, p=0.027)	2.9 (1.1 – 8.1, p=0.031)
Dyspnea	22 (14.5%)	29 (23.0%)	1.8 (0.9 – 3.3, p=0.07)	1.8 (0.9 – 3.3, p=0.07)
Chest pain (%)	52 (34.2%)	40 (31.8%)	0.9 (0.5 – 1.5, p=0.66)	0.9 (0.5 – 1.4, p=0.60)
Dizziness (%)	34 (22.4%)	32 (25.4%)	1.2 (0.7 – 2.1, p=0.56)	1.2 (0.7 – 2.0, p=0.59)
Fatigue (%)	9 (5.9%)	6 (4.8%)	0.8 (0.3 – 2.3, p=0.67)	0.8 (0.3 – 2.5, p=0.72)
Headache (%)	7 (4.6%)	7 (5.6%)	1.2 (0.4 – 3.6, p=0.72)	1.2 (0.4 – 3.5, p=0.77)
Loss of consciousness (%)	11 (7.2%)	4 (3.2%)	0.4 (0.1 – 1.4, p=0.15)	–
Presyncope (%)	6 (4.0%)	4 (3.2%)	0.8 (0.2 – 2.9, p=0.73)	–
Syncope (%)	1 (0.7%)	1 (0.8%)	1.2 (0.1 – 19.5, p=0.89)	–
Vomiting (%)	1 (0.7%)	2 (1.6%)	2.4 (0.2 – 27.2, p=0.47)	–
Panic (%)	7 (4.6%)	7 (5.6%)	1.2 (0.4 – 3.6, p=0.72)	1.1 (0.4 – 3.3, p=0.84)

All results are adjusted for potential confounders such as age, Charlson Co-morbidity index and the need for any treatment for small narrow tachycardia in the past.

### 3.3 Diagnostics

There was no sex-specific difference in the waiting time (adj. difference 2.0 (95% CI -3.6 – 7.6, p=0.48)) until the first medical contact and there was also no difference in the duration of treatment (adj. difference 14.7 (95% CI -43.9 – 14.5, p=0.32)) between the sexes (**Table 4**). All patients received an ECG (n= 278, 100%) and a blood sample (n=278, 100%) on ED admission. In the blood analysis, a significantly increased frequency of hypokalemia in women (11.1% vs. 3.3%, adj. OR 3.7 (95% CI 1.3 – 10.9, p=0.018)) was detected. Additionally, there was no association between hypokalemia with nausea (OR 1.6, 95% CI 0.4 – 6.5, p=0.53), anxiety (OR 3.6, 95% CI 0.6 – 21.9, p=0.17) and palpitation (OR 0.8, 95% CI 0.1 – 7.2, p=0.69) in women, therefore symptoms as nausea, anxiety, or palpitations were not caused by hypokalemia. In the majority of all patients (88.9%, n = 247), high-sensitive troponin was analyzed, whereas 22.7% (n = 56) of these patients had a one-hour follow-up. In 11 patients (4%), a transthoracic echocardiography (TTE) was performed by consulted cardiologists.

**Table 4:** ED indicators, diagnostic tests and the results in the ECG.

Diagnostic tests	All patients n = 278	Male patients n = 152 (54.7%)	Female patients n = 126 (45.3%)
Waiting time in the ED (min)	11.5 (5 – 24)	11 (4.5 – 24.5)	12 (5 – 24)
Treatment time in the ED (min)	233 (165 – 323)	235.5 (174.5 – 335)	221.5 (154 – 319)
Serum blood analysis (%)	278 (100%)	152 (100%)	126 (100%)
TTE FAST (%)	3 (1.1%)	2 (1.3%)	1 (0.8%)
TTE by cardiologist (%)	11 (4.0%)	6 (4.0%)	5 (4.0%)
ECG (%)	278 (100%)	152 (100%)	126 (100%)
- Atrial fibrillation (%)	52 (18.7%)	31 (20.4%)	21 (16.7%)
- Sinus tachycardia (%)	41 (14.8%)	21 (13.8%)	20 (15.9%)
- Atrioventricular nodal reentrant tachycardia (AVNRT) (%)	25 (9.0%)	9 (5.9%)	16 (12.7%)
- Atrial flutter (%)	21 (7.6%)	17 (11.2%)	4 (3.2%)
- Ventricular extrasystoles (VES) (%)	15 (5.4%)	12 (7.9%)	3 (2.4%)
- Supra-ventricular extrasystoles (SVES) (%)	8 (2.9%)	7 (4.6%)	1 (0.8%)
- Atrial tachycardia (%)	6 (2.2%)	2 (1.3%)	4 (3.2%)
- Supraventricular tachycardia not classified (%)	5 (1.8%)	2 (1.3%)	3 (2.4%)
- Atrioventricular reentrant tachycardia (AVRT) (%)*	4 (1.4%)	2 (1.3%)	2 (1.6%)
- Focal atrial tachycardia (%)	1 (0.4%)	1 (0.7%)	0%
- Junctional ectopic tachycardia (%)	1 (0.4%)	0%	1 (0.8%)
- Multifocal atrial tachycardia (%)	0%	0%	0%

TTE = transthoracic echocardiography; Results were presented as median (25th – 75th percentile).

ECG = electrocardiogram TTE = transthoracic echocardiography; Results were presented as median (25th – 75th percentile).

* Including Wolff Parkinson White.

Across all patients, AF was the most common diagnosis, followed by sinus tachycardia (**Table 4**). AVNRT was significantly more frequent in women (12.7% vs. 5.9%, adj OR 2.4 (1.01 – 5.8, p=0.047), whereas atrial flutter was significantly less common (3.2% vs. 11.2%, adj. OR 0.2 (0.07 – 0.7, p=0.011). An overview of all rhythm diagnoses and sex-specific distributions is provided in **Table 4**.

### 3.4 Treatment & follow-up care

There was no relevant sex-specific difference in the choice or success of treatment procedures (**Supplementary Table 1**). Over two-thirds of patients did not require any therapeutic intervention due to self-limitation of the symptoms. The most common medical treatments were administration of beta-blockers and adenosine, followed by modified vagal manoeuvres. Electrical cardioversion and amiodarone were rarely used (**Supplementary Table 1**).

Furthermore, no significant sex-specific differences were found in the follow-up treatment (**Table 5**). A cardiological evaluation in the ED was carried out in a total of 105 patients (27.8%). In 64.4% (n= 179), a cardiological out-patient follow-up was performed, and in 24.1% such an assessment was at least recommended. Similarly, the need for ablation remained consistent across all patients (**Table 5**).

**Table 5:** Sex differences in the outcome.

Outcome	Male patients n=152 (54.7%)	Female patients n=126 (45.3%)	Unadjusted OR (95% CI, p-value)	Adjusted OR (95% CI, p-value)
Co-evaluation by cardiologists in the ED (%)	65 (42.8%)	40 (31.8%)	0.6 (0.4 – 1.0, p=0.06)	0.6 (0.4 – 1.1, p=0.08)
Outpatient cardiac assessment during the course (%)	103 (67.8%)	76 (60.3%)	0.7 (0.4 – 1.2, p=0.20)	0.7 (0.4 – 1.2, p=0.24)
Recommendation for cardiac assessment by ED (%)	34 (22.4%)	33 (26.2%)	1.2 (0.7 – 2.1, p=0.46)	1.3 (0.3 – 5.2, p=0.74)
Need of ablation during the course (%)	20 (13.2%)	20 (15.9%)	1.4 (0.8 – 2.5, p=0.18)	1.5 (0.8 – 2.6, p=0.19)
Recurrence after index ED discharge	6 (4.0%)	11 (8.7%)	2.3 (0.8 – 6.5, p=0.11)	2.4 (0.8 – 6.8, p=0.10)
Outcome			Unadjusted difference (95% CI, p-value)	Adjusted difference (95% CI, p-value)
Ablation after X days after index ED discharge	53 (22 – 144)	108 (40 – 153)	46.9 (-59.3 – 153.0, p=0.38)	46.5 (-59.3 – 152.3, p=0.38)
Recurrence after X days after index ED discharge	40.5 (9 – 83)	54 (4 – 140)	27.1 (-55.4 – 109.5, p=0.50)	21.0 (-58.7 – 100.8, p=0.58)

All results are adjusted for potential confounders such as age, Charlson Co-morbidity index and the need for any treatment for small narrow tachycardia in the past.

ED, Emergency Department.

## 4 Discussion

A growing amount of sex-based research is providing insights into sex-specific differences in various cardiovascular diseases. In our study, we investigated potential sex-specific differences from ED patients presenting with SVT in a tertiary care hospital. While symptom onset was sudden in both sexes and most episodes were self-limiting regardless of sex, women reported a significantly higher number of symptoms compared to men. Women developed more likely palpitations, nausea, and anxiety than male patients. A trend toward more frequent reporting of dyspnea among women was also observed, although this did not reach statistical significance. Despite these differences in symptom presentation, our study found no significant sex-specific differences in the extent of diagnostic procedures performed in the ED. Regarding the final diagnosis, AVNRT was diagnosed significantly more often in women, while atrial flutter was identified more frequently in men. There were no statistically significant differences in objective treatment modalities between sexes. However, since we did not assess patients’ subjective treatment experiences, no conclusions can be drawn regarding whether women experienced their concerns as being taken as seriously as those of men.

Taken together, these findings support the significance of considering sex-specific factors in both the diagnosis and management of supraventricular tachycardias.

### 4.1 Prevalence patterns of SVT

The literature shows a twofold higher risk of SVT for women than for men (2, 3, 7). In our study, we had an approximately equal sex distribution, but we included both AF and atrial flutter in the definition of SVT. Both diagnoses are more common in men, which may explain the equal distribution in our study. In our study, male patients exhibited higher levels of alcohol consumption compared to female patients, which is consistent with existing literature indicating that men still account for a greater proportion of total alcohol consumption and related adverse outcomes, although this gap is gradually narrowing (15). While the association between alcohol consumption and SVTs in general remains incompletely understood, alcohol is a well-established trigger for AF (16), suggesting a potential contributory role of alcohol in the observed sex distribution in our cohort. Comparable to the literature, we could also show that women are significantly more likely to have AVNRT (2, 6). The reason for this predisposition for AVNRT in female can be explained by a hormonal connection and thus a relationship with the woman’s menstrual cycle (9, 17). In line with this, an increased frequency of episodes during pregnancy in women with pre-existing AVNRT has also been described (2, 6, 7). Additionally, it is known that women are more frequently affected by atrial tachycardia compared to men (11, 18), whereas men are more prone to AVRT (11) and atrial flutter (2). In our study, atrial flutter was also significantly more often seen in men than in female ED patients. Concerning AVRT in the current study, both patients suffering from AVRT were male. Based on our investigation, it can be tentatively concluded that AF was the most common diagnosis in all sexes, followed by sinus tachycardia. Subsequently, AVNRT ranked third among women, while atrial flutter ranked third among men. Similarly, the same can be said regarding inappropriate sinus tachycardia, which is frequently reported in the literature as being more prevalent in women (19, 20). However, no difference was observed in our cohort. This observation may be confounded by the fact that a high number of the current patients already reverted to sinus rhythm upon presentation to the ED, obscuring the accurate diagnosis.

### 4.2 Symptom presentation in SVT

Previous literature describes that women experience more symptoms during SVT episodes (10, 11), yet it remains unclear whether this reflects greater symptom burden or simply a higher number of reported symptoms. In the current study, women described a significantly broader range of symptoms per episode. Possible explanations include a greater tendency among women to verbalize symptoms, hormonal influences on symptom perception, and sex-specific differences in autonomic regulation, particularly the more pronounced parasympathetic activity described in women (21–23).

Furthermore, various studies have described sex-specific differences in symptom presentation: women expressed more commonly anxiety and palpitations, particularly in the context of SVT (4, 7, 24), including atrial fibrillation (18, 25). Additionally, several investigations have indicated that women experienced a poorer quality of life (24), with these symptoms often interpreted within the context of anxiety and panic disorders (7), leading to an increased risk of misdiagnosis (24). Our study confirms these findings, as women also reported significantly more frequent palpitations and anxiety, in addition with a significantly higher incidence of nausea. Previous literature also indicates that women with symptoms were not taken seriously by clinicians, contributing to diagnostic delays and suggesting potential bias in clinical practice (6, 7, 10). As our study was based on retrospective review rather than patient interviews, we cannot directly assess perceived diagnostic bias. However, the higher prevalence of symptoms previously associated with misinterpretation highlights the need to consider arrhythmia in the differential diagnosis and to obtain an electrocardiogram in patients presenting with palpitations, anxiety or nausea.

Dyspnea, which has been noted as more characteristic for women in other studies (7), did not exhibit a significant difference in the current analysis, although a trend was discernible. This may be due to limited sample size or the fact that many patients had already converted upon ED admission, potentially reducing the accuracy of symptom reporting.

On the other hand, palpitations have been described as the most frequent symptom leading to invasive evaluation (26), which aligns with our finding that palpitations were also the most commonly reported symptom. In contrast to literature in which women presented with higher heart rates (20), no difference in the median heart rate between the sexes could be identified in our study. However, the bias cannot be ruled out here either, as most patients were already in sinus rhythm when they arrived at the ED. In contrast to prior reports of higher rates of ED visits and hospital admissions among women with SVT (7), we did not observe a higher rate of readmissions for similar complaints in our cohort.

Electrolyte disturbances, including hypokalemia, are known triggers for SVT. Alcohol consumption has likewise been described as a potential precipitating factor; however, while the association is well established for AF, the evidence for other forms of SVT remains less consistent and in part controversial (16, 27). In our study cohort, hypokalemia was significantly more prevalent among women, whereas increased regular alcohol consumption was significantly more common among men. This distribution is consistent with epidemiological data demonstrating a higher risk of hypokalemia among women and a higher overall alcohol consumption among men (15, 28). In our cohort, a subset of patients was receiving thyroid hormone replacement therapy. Overtreatment with levothyroxine may induce a state of (subclinical) hyperthyroidism, which has been associated with an increased risk of cardiac arrhythmias, particularly atrial fibrillation, with higher susceptibility reported in older individuals and in the presence of suppressed TSH levels (29, 30). Only a small number of patients in our cohort were diagnosed with hyperthyroidism, and among these, thyroid dysfunction was not consistently related to levothyroxine therapy, as only one patient was receiving thyroid hormone replacement, while another was treated with antithyroid medication. Therefore, its contribution to the observed arrhythmia patterns in our cohort is likely limited.

### 4.3 Approaches to diagnosis and management of SVT

Based on the diagnostics performed in the ED, we were unable to identify any sex-specific differences. Fortunately, this also applies to the type or success of treatment in the ED. Similarly, there was no difference regarding a co-evaluation carried out by the cardiologist in the ED or any cardiological follow-up checks that were carried out or recommended.

Concerning treatment, the literature describes that women were more frequently treated primarily with medication instead of ablation (2, 6–8, 26). Additionally, lower rates of ablation were generally observed in women compared to men (7), and if performed, it was done at a later stage (6, 8, 10, 11, 25, 26, 31). In our study, we did not find any difference in the ablation rate between the sexes. However, as a limitation of this current study, as we did not carry out a follow-up, we do not know how many patients may have had an external ablation after the index ED visit, which could therefore bias the results. Based on the data in our patient records, it can be seen that female and male patients have undergone ablation to a similar extent in the past. In the literature, we encountered diverse findings regarding the recurrence rate, with some studies reporting it being higher in women (2), while others describe it as equivalent (6, 18, 24). However, there are accounts suggesting that women may suffer from symptoms for a longer duration post-ablation compared to men (6, 10, 18). Nevertheless, it has been deemed safe and successful for both sexes (6, 11, 18, 24). In our cohort, we also found no sex-specific differences in the rate of readmission after ablation. However, it’s again worth noting that we lack follow-up data, therefore we do not have information regarding potential visits to external physicians or other hospitals.

### 4.4 Limitations

A strength of the study is our homogeneous distribution of sexes, which contrasts with many other studies, and the inclusion of confounders in the regression analysis. Limitations include the lack of randomization, the absence of follow-up data, and the fact that it is a single-centre study. Nevertheless, it should be noted that this study was conducted in a university hospital setting.

## 5 Conclusion

In conclusion, women presenting with SVT often express symptoms more typical for their gender, which may closely resemble those of anxiety or panic disorders. Evidently, these symptoms in women are frequently misinterpreted within the context of such disorders, leading to delayed diagnosis and increased risk for further complications. It is imperative to recognize this bias in the management of women presenting with symptoms such as palpitations, anxiety, and nausea, and to ensure appropriate diagnostic work-up for SVT. Given the risk of misattribution to non-cardiac causes, performing an ECG - particularly in women presenting with symptoms such as panic, anxiety, or nausea - is essential to avoid missing arrhythmic events.

More broadly, such misattribution is not exclusive to women. A low threshold for obtaining an ECG is warranted in all patients presenting with compatible symptoms, regardless of sex—particularly when palpitations, anxiety, panic-like symptoms, or nausea are reported. This proactive approach ensures timely identification of SVT and prevents missed or delayed diagnoses of clinically relevant arrhythmias.

## Data Availability

The data analyzed in this study is subject to the following licenses/restrictions: Data are in the EHR of University Hospital of Zurich. Requests to access these datasets should be directed to ksenija.slankamenac@usz.ch.
