# Supplementary material for: Recognizing supraventricular tachyarrhythmias in female patients: beyond palpitations to symptoms like anxiety, panic, and nausea
Source: Front Psychiatry. 2026 Jul 22;17:1858764. doi: 10.3389/fpsyt.2026.1858764 (PMC13438066; doi:10.3389/fpsyt.2026.1858764)
Supplement: Supplementary file 1 [file Table1.docx]

Supplementary Material

**Supplemental Table**

**Table S1** Treatment approaches

|  | **All patients**  n=278 | **Male patients**  n=152 (54.7%) | **Female patients**  n=126 (45.3%) |
| --- | --- | --- | --- |
| Beta-blocker treatment (%)   - total doses [mg] - successful=rhythm control - heart rate control only | 49 (17.6%)  5 (2.5 – 5)  15  11 | 30 (19.7%)  5 (2.5 – 5)  10  6 | 19 (15.1%)  2.5 (1.25 – 5)  5  5 |
| Adenosine application intravenous (%)   - number of adenosine doses - successful after one dose iv - successful after two doses iv - successful after more than two doses - no success | 26 (9.4%)  1 (1 – 2)  12  3  2  9 | 13 (8.6%)  1 (1 – 2)  5  1  1  6 | 13 (10.3%)  1 (1 – 2)  7  2  1  3 |
| Modified vagal maneuver in the ED (%)   - successful | 18 (6.5%)  2 | 10 (6.6%)  2 | 8 (6.4%)  - |
| Amiodaron treatment (%)   - maintenance therapy iv (%) - successful | 8 (2.9%)  0%  4 (1.4%) | 7 (4.6%)  0%  4 (2.6%) | 1 (0.8%)  0%  0% |
| Electro conversion (%)   - after 1^st^ (120 Joule) - after 2 - (200 Joule) | 6 (2.2%)  4  2 | 3 (2.0%)  3  - | 3 (2.4%)  1  2 |
| Vagal maneuver, self-employed (%)   - successful | 6 (2.2%)  2 | 3 (2.0%)  - | 3 (2.4%)  2 |
| Drinking ice water (%)   - successful | 4 (1.4%)  - | 2 (1.3%)  - | 2 (1.6%)  - |
| Vagal maneuver in the ED (%)   - successful | 7 (2.5%)  1 | 3 (2.0%)  - | 4 (3.2%)  1 |
| Diltiazem treatment (%) | 0% | 0% | 0% |
| Verapamil treatment (%) | 0% | 0% | 0% |
| Flecainide treatment (%)   - successful | 3 (1.1%)  2 | 2 (1.3%)  1 | 1 (0.8%)  1 |
| Need for noradrenaline (%) | 0% | 0% | 0% |
| *ED = Emergency Department; Results were presented as median (25th – 75th percentile)* | | | |
